# Analysis of Survival Curves: Statistical Methods Accounting for the Presence of Long-Term Survivors

**DOI:** 10.3389/fonc.2019.00453

**Published:** 2019-06-04

**Authors:** Vera Damuzzo, Laura Agnoletto, Luca Leonardi, Marco Chiumente, Daniele Mengato, Andrea Messori

**Affiliations:** ^1^Department of Pharmaceutical and Pharmacological Sciences, School of Hospital Pharmacy, University of Padua, Padua, Italy; ^2^Hospital Pharmacy, Hospital of Rovigo, AULSS 5 Polesana, Rovigo, Italy; ^3^Department of Pharmacy, Post Graduate School of Hospital Pharmacy, University of Pisa, Pisa, Italy; ^4^Scientific Direction, Italian Society for Clinical Pharmacy and Therapeutics, Milan, Italy; ^5^Hospital Pharmacy, Bolzano Central Hospital, Bolzano, Italy; ^6^HTA Unit, Regional Health Service, Florence, Italy

**Keywords:** survival, adult, Kaplan-Meier estimator, survival plateau, area under the curve, median survival

## Abstract

Some anti-cancer treatments (e. g., immunotherapies) determine, on the long term, a durable survival in a small percentage of treated patients; in graphical terms, long-term survivors typically give rise to a plateau in the right tail of the survival curve. In analysing these datasets, medians are unable to recognize the presence of this plateau. To account for long-term survivors, both value-frameworks of ASCO and ESMO have incorporated *post-hoc* corrections that upgrade the framework scores when a survival plateau is present. However, the empiric nature of these *post-hoc* corrections is self-evident. To capture the presence of a survival plateau by quantitative methods, two approaches have thus far been proposed: the milestone method and the area-under-the-curve (AUC) method. The first approach identifies a long-term time-point in the follow-up (“milestone”) at which survival percentages are extracted. The second approach, which is based on the measurement of AUC of survival curves, essentially is the rearrangement of previous methods determining mean lifetime survival; similarly to the milestone method, the application of AUC can be “restricted” to a pre-specified time-point of the follow-up. This Mini-Review examines the literature published on this topic. The main characteristics of these two methods are highlighted along with their advantages and disadvantages. The conclusion is that both the milestone method and the AUC method are able to capture the presence of a survival plateau.

## Introduction

In the past 5 years, a renewed interest has been focused on qualitative methods that grade the clinical value of anti-cancer treatments. These methods, that are frequently denoted as value frameworks, have been widely debated in the oncologists' community, and their advantages and disadvantages have been highlighted (1–3). Two main frameworks have emerged in this context: the ASCO value framework (4) developed by the American Society of Clinical Oncology and the ESMO Magnitude of Clinical Benefit scale (5), developed by the European Society of Medical Oncology. Both methods generate a qualitative score: the ASCO score ranges from 0 to 100 while the ESMO one ranges from 1 to 5 (and from C to A for curative treatments); the higher the score, the better the clinical value of the treatment concerned. The ASCO score should be seen as a qualitative method, not a quantitative one, because it synthetizes a series of qualitative assessments concerning incremental benefit and toxicity and also because it is not based on any units of measurements.

More recently, the demonstration that some anti-cancer treatments (e.g., immunotherapies) provide a durable survival in a small percentage of treated patients (6, 7) has raised a difficult methodological question: in fact, these long-term survivors determine a survival plateau in the right tail of the Kaplan-Meier curve, but in analysing these datasets medians are unable to recognize the presence of this plateau. In both approaches of ASCO and ESMO, *post-hoc* corrections of the scores (8–10) have been proposed whereby scores are upgraded when a survival plateau is present. However, the empiric nature of these *post-hoc* corrections is self-evident.

In this context, the need to develop quantitative methods that capture the presence of a survival plateau has emerged. Thus, far, two quantitative methods have been proposed: the milestone method (11–13) and the AUC method (14). The first method requires to identify a “long-term” time-point in the survival curve (called “milestone”) that must be longer than median survival; then, the quantitative index of the analysis is directly represented by the survival percentage at the pre-determined milestone. The second method, which has only been reported in abstract form (14), is the rearrangement of a well-known approach based on mean lifetime survival that has been used for many decades (15, 16). The main advantage of these two methods lies in their ability to capture the presence of a survival plateau and to consequently quantify the “weight” of the plateau within the whole shape of the survival curve.

Table 1 presents a glossary that explains the main parameters employed in the analysis of survival curves.

**Table 1:** Glossary of the main technical parameters.

**Parameter**	**Abbreviation**	**Units**	**Meaning**
Hazard ratio	HR	Adimensional	The event risk in the comparison between the experimental group and the control group
Mean survival time	MST	Time	The survival estimate per patient from time 0 to infinity; MST is calculated from the area under the survival curve, using a model-based estimation method (e.g., proportional hazard model, models of Weibull, Gomperz, etc.)
Restricted mean survival time	RMST	Time	The survival estimate per patient calculated from time 0 to a pre-determined time-point in the follow-up; calculations are the same as those employed for MST
Mean lifetime survival	MLS	Time	Synonymous for MST
Area under the survival curve	AUC	Time	The same as MST, but has a model independent nature because of its estimation according to the trapezoidal rule
Restricted area under the survival curve	rAUC	Time	The same as AUC, but is calculated from time 0 to a pre-determined time-point
Milestone survival	–	Percentage (from 0 to 100%) or rate (from 0 to 1)	The survival value calculated at a predetermined time-point according to the Kaplan-Meier curve

## The Milestone Method

Evaluations of survival at “milestones” is the analysis of survival rates at a fixed time point of follow-up. This approach has been proposed as a means of capturing the right tail of long-term survivors (11, 13) even though, in some applications, also short-term milestones have been employed.

In more detail, milestone survival is defined as the Kaplan-Meier survival probability at a time point defined a priori (e.g., 60 months). Milestone survival analysis is a cross-sectional assessment of the survival data at the prespecified time point using Kaplan-Meier probabilities. The choice of the milestone requires careful consideration because it often represents a clinically meaningful benchmark. It is important to stress that the milestone does not necessarily represent long-term survival. It may represent a time point beyond which the treatment benefit is thought to remain stable. To determine the time point of interest, sufficient follow-up duration is generally required to contribute enough information to the milestone analysis.

An advantage of the milestone method lies in its intuitive ability of easily describing the presence of durable survival. However, milestones have an intrinsic limitation because they depend on the specific time point chosen for the analysis. Hence, unlike medians, milestones cannot be generalized when different treatments aimed at different disease conditions are best described by different milestone time points.

The milestone method has two different purposes: (a) in the analysis of randomized controlled trials, the ratio between the milestone rate in the treatment group and the milestone rate in the controls captures the incremental effect of the experimental treatment (particularly when treated patients have durable survival unlike the controls, but medians are similar across the two patient groups); (b) in the analysis of one-arm trials, the milestone rate compares different treatments across different trials studying the same disease condition and is typically focused on long term effects especially when medians are similar to one another and do not capture the tail of the survival curves.

## The AUC Method

The ratio between the area under the survival curve (AUC) and median survival has been proposed as a new parameter to capture the presence of a plateau in survival curves (14). The AUC (as well as its proxy represented by the mean lifetime survival) has already been described many years ago (15, 16). Nowadays, its practical calculation is facilitated by the availability of websites that handle the mathematics of graphical curves.

Mean lifetime survival differs from restricted AUC (rAUC) because rAUC is calculated from time zero until the last time-point in the follow-up, whereas mean lifetime survival includes the extrapolation of survival from the last time-point to infinity, e.g., according to the equations of Gompertz or Weibull (15, 16). One advantage of both AUC and rAUC is that, like in traditional pharmacokinetic analyses (17, 18), the trapezoidal rule permits to reliably estimate their values without any mathematical complexity. Despite the different purposes, survival analysis (handled through AUC) and pharmacokinetic analysis share exactly the same theory and the same computational tools.

Like the milestone method, also the AUC method has two different purposes: (a) in the analysis of randomized controlled trials, the ratio between the AUC values in the treatment group and in the controls resembles the hazard ratio, and therefore tends to reassess the information provided by the hazard ratio; (b) in the analysis of one-arm trials, the ratio between AUC and median is calculated; a long-term survival plateau is shown by the finding that the AUC is greater than the median (ratio > 1); the more this ratio is greater than 1, the greater the “impact” that long-term survivors have on the entire survival pattern. This numerical property of the ratio AUC/median reflects an intuitive concept because a survival plateau is always a prolongation of survival in comparison with the absence of a plateau.

One advantage of AUC lies in its computational simplicity owing to the model-independent nature [as opposed to other methods, e.g., those of Gompertz and Weibull, that are model-dependent and require a complex mathematical analysis (15, 16)]. Another strength is in operational terms because specific references are available about the software tools that apply this method (see Appendix).

On the other hand, the ratio AUC/median retains a limitation of median in that this new parameter cannot be calculated when residual survival is more than 50%; in these cases, however, determining the presence of a plateau in the right tail of the curve makes little sense.

Finally, it should be noted that, in analysing a single survival curve, the AUC is equivalent to the parameter called mean survival time (MST); in more detail, AUC_0−>last time point_ is identical to restricted MST (RMST), while AUC_0−>infinity_ is identical to (unrestricted) MST, otherwise denoted as mean lifetime survival (MLS). In the past, model-dependent methods have typically been used for the mathematical computation of MST, RMST, MLS from the curve; in the present paper, AUC is intended to be estimated by model-independent methods.

## Comparative Performance Between the Milestone Method and the AUC Method

We have re-analyzed the two data sets presented by Hellman et al. (12) to describe their approach based on milestones: (1) Comparison of progression-free survival between gefitinib vs. paclitaxel+carboplatin in 1,217 patients with advanced pulmonary adenocarcinoma; (2) Comparison of overall survival between ipilimumab+dacarbazine vs. placebo+dacarbazine in 60 patients with advanced melanoma. The first dataset was proposed by Hellman et al. as a typical example where “the median is the message” (12); the second was as an example where “the milestone is the message” (12).

**Figure 1:**
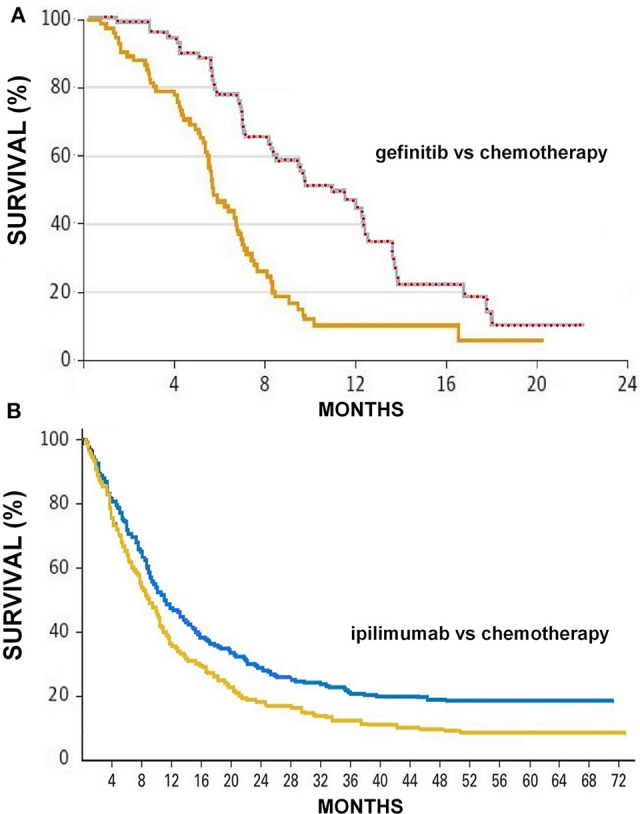
**(A)** Progression-free survival curves reported by Fukuoka et al. (21) in patients treated with gefinitib (upper curve) or chemotherapy (lower curve); the series of red circles shows how the automated digitalizer identified the points that define the upper boundary of the area under the curve comprised between 0 and 23 months (area = 11.31 months). **(B)** Overall survival curves reported by Maio et al. (22) in patients treated with ipilimumab (upper curve) or chemotherapy (lower curve); also these two curves were analyzed by the automated analyser to determine the two values of area under the curve. Survival expressed in percentage and time in months.

Table 2 summarizes the results obtained by analysing these two data sets according to both the milestone method and the AUC method. Figure 1 shows how the 4 curves were digitalized for the calculation of AUC.

**Table 2:** Results of the comparison of performance between the milestone method and the AUC method.

**Trial**	**End-point**	**Median (months)**		**Milestone survival rate (%)**		**AUC (months)**	
		**Experimental arm**	**Control arm**	**Experimental arm**	**Control arm**	**Experimental arm**	**Control arm**
Gefitinib vs. paclitaxel + carboplatin in patients with metastatic non-small-cell lung cancer	Progression-free survival	9.1	5.4	65.8% at 8 months	25.9% at 8 months	11.3^*^	6.86^*^
Ipilimumab + dacarbazine vs. placebo + dacarbazine in patients with metastatic melanoma	Overall survival	11.2	9.1	18.2% at 5 years	8.8% at 5 years	23.1^**^	16.1^**^

* The ratio of the two values of AUC (0.61) is not similar to the hazard ratio reported in the original trial (0.38; 95% CI, 0.26–0.56).

** The ratio of the two values of AUC (0.70) is similar to the hazard ratio reported in the original trial (0.69; 95% CI, 0.57–0.84).

*All survival information was drawn from the article by Hellman et al. (12); the values of median survival were those of the original trials*.

Although these two methods pursue a similar objective, their results are clearly not comparable with one another. This is because the milestone method and the AUC methods are based on two different perspectives of analysis that are incompatible.

In the milestone analysis of the first data set, placing the milestone between the values of the two medians (i.e., at 8 months) maximized the magnitude of the survival difference expressed in percentage points; in this case “the median is the message,” and in fact medians adequately represented the better result obtained in the experimental arm. As indicated by the shape of the two curves, placing the milestone at a longer follow-up in this dataset would not provide any advantage (data not shown).

In the AUC analysis of the first dataset, the most interesting finding is that the ratio AUC/median was 1.24 in the gefitinib arm vs. 1.27 in the paclitaxel+carboplatin arm. Although, as expected, AUC was longer than median in both arms, the former exceeded the latter by <30% (relative difference). This finding supports the conclusion that the median is the message. In this dataset, the ratio of the two AUC was not similar to the hazard ratio reported in the trial owing to reasons that are difficult to explain; compared with the shape of the two curves, the hazard ratio of 0.38 seems in fact to be excessively favorable.

In the milestone analysis of the second data set, placing the milestone on the long term (i.e., at 60 months) maximized the magnitude of the survival difference expressed in percentage points; in this case “the milestone is the message,” and in fact milestones adequately represented the better long-term result obtained in the experimental arm (survival plateau) compared with the controls. As indicated by the shape of the two curves, placing the milestone at a long follow-up produced a more meaningful clinical result than that indicated by medians.

In the AUC analysis of the second dataset, the most interesting finding is that the ratio AUC/median was 2.06 in the ipilimumab+dacarbazine arm vs. 1.77 in the placebo+dacarbazine arm. These findings, where AUC tends to be approximately twice the median, are in keeping with the conclusion that the median is not the message. In this dataset, the ratio of the two AUC was similar to the hazard ratio.

## Discussion

Capturing a survival plateau can be useful for a variety of reasons. For many decades, median has been the standard parameter for summarizing outcomes in oncology, and its role is undisputed owing to the long-standing scientific reputation and ubiquitous use. Despite this, the availability of additional parameters for analysing survival curves (such as the milestone or the ratio AUC/median) can be justified to fill the gap represented by the inability of medians to account for the final portion of survival curves.

A preliminary experience has already accumulated on the milestone method whereas less experience is available with the AUC method. In particular, the ratio between AUC and median seems to be a parameter of remarkable interest, even though a more thorough confirmation will be required based on an adequate number of survival curves.

Finally, considerable literature has accumulated in the past years about other innovative statistical methods that can improve the interpretation of survival curves (23–25). Since the approach based on RMST is the one most commonly employed (23, 25), some specific comments on this technique are warranted. In comparing two survival curves (experimental arm vs. control arm), the RMST, which can be considered an area under the survival curve, can be used either as the ratio of two RMSTs (i.e., RMST_experimental_/RMST_control_) or their difference (i.e., RMST_experimental_ - RMST_control_). In the first case (23, 24), the ratio is adimensional (like the HR) and in fact can essentially be interpreted as an HR (without any substantial difference). In the second case (23), the RMST difference has the units of time (like the difference of the two medians) and can be seen as an improved estimate of the survival gain. This is because the RMST is influenced by the entire shape of the survival curve whereas the median has the drawback of being a punctiform parameter that does not reflect the whole survival curve.

One important point is that the RMST has been developed as a tool that quantitatively improves the representation of survival curves but not as a parameter that captures the presence of long-term survivors. In contrast, both the milestones method (12) and our method based on the ratio AUC/median (14) have been specifically designed to capture the presence of a long-term survival plateau in the right tail of the curve. This is the reason why the present review has been focused more on milestones and on the ratio AUC/median than on the RMST.

The recent paper published by Wang et al. (25) has combined the advantages of milestones and of RMST, but unfortunately the ratio of the two RMSTs has been expressed in an atypical reciprocal form, that prevents a sound interpretation of the findings published by these authors (26, 27).

## Author Contributions

All authors listed have made a substantial, direct and intellectual contribution to the work, and approved it for publication.

### Conflict of Interest Statement

The authors declare that the research was conducted in the absence of any commercial or financial relationships that could be construed as a potential conflict of interest.

## Appendix

In the application of the AUC method, the survival curves can be digitalized using a web-based program (19); in this phase of the analysis, the above software identifies (for each survival curve) the y-vs.-x data pairs describing the Kaplan-Meier graph. Thereafter, the AUC can be calculated for each curve using the trapezoidal rule, as in pharmacokinetics (17). A subroutine written in Microsoft Excel is available for this purpose (20).
